# Supplementary figures and images for: Future climate-driven habitat loss and range shift of the Critically Endangered whitefin swellshark (Cephaloscyllium albipinnum)
Source: PeerJ. 2025 Feb 20;13:e18787. doi: 10.7717/peerj.18787 (PMC11847489; doi:10.7717/peerj.18787)

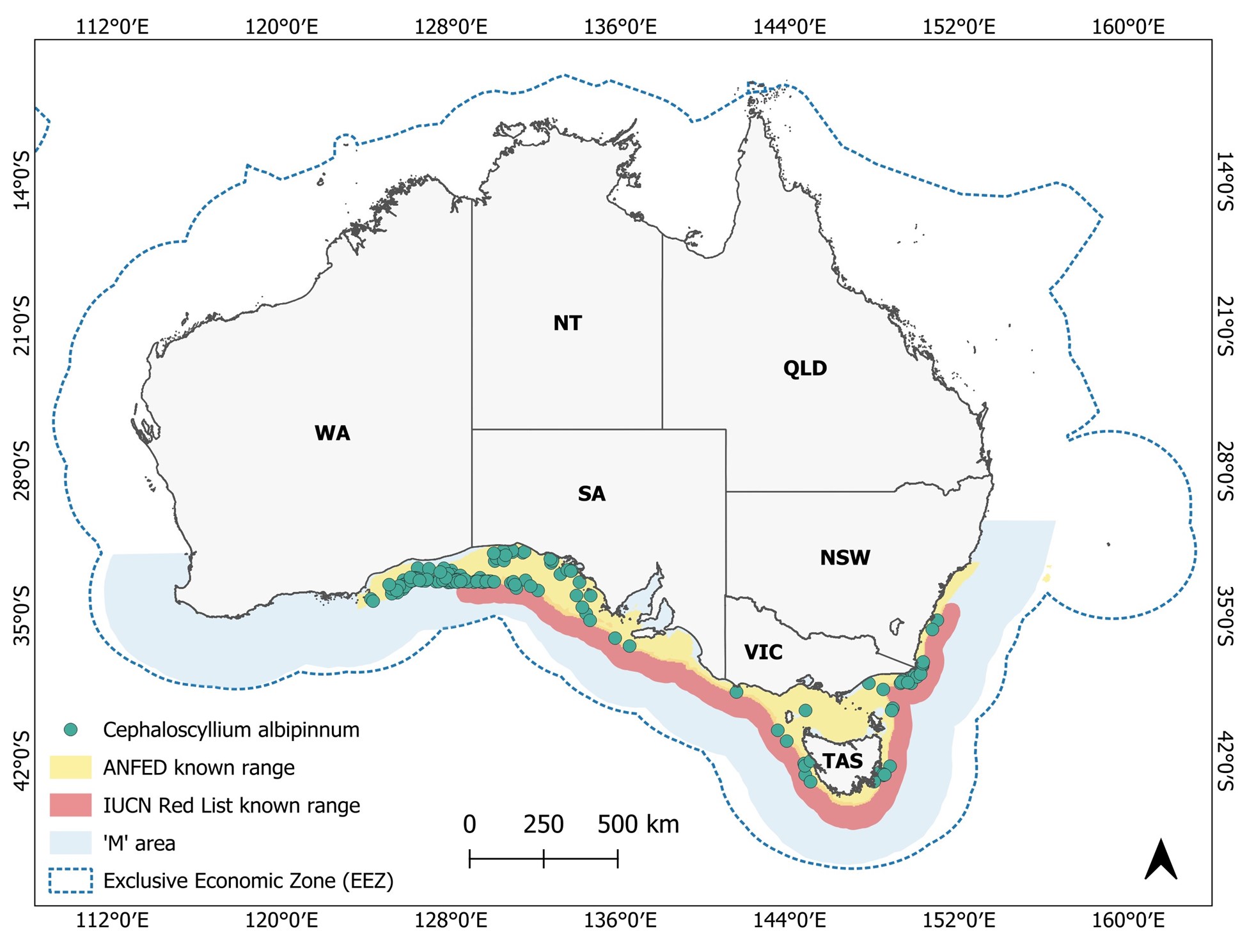

Supplement: Supplemental Information 1 — The known range as acknowledged by the Australian National Fish Expert Distribution (yellow area; CSIRO Marine and Atmospheric Research, 2012) and the International Union for Conservation of Nature Red List (orange area; International Union for Conservation of Nature, 2012) are indicated. The blue area represents the calibration region (M area). [file peerj-13-18787-s001.jpg]

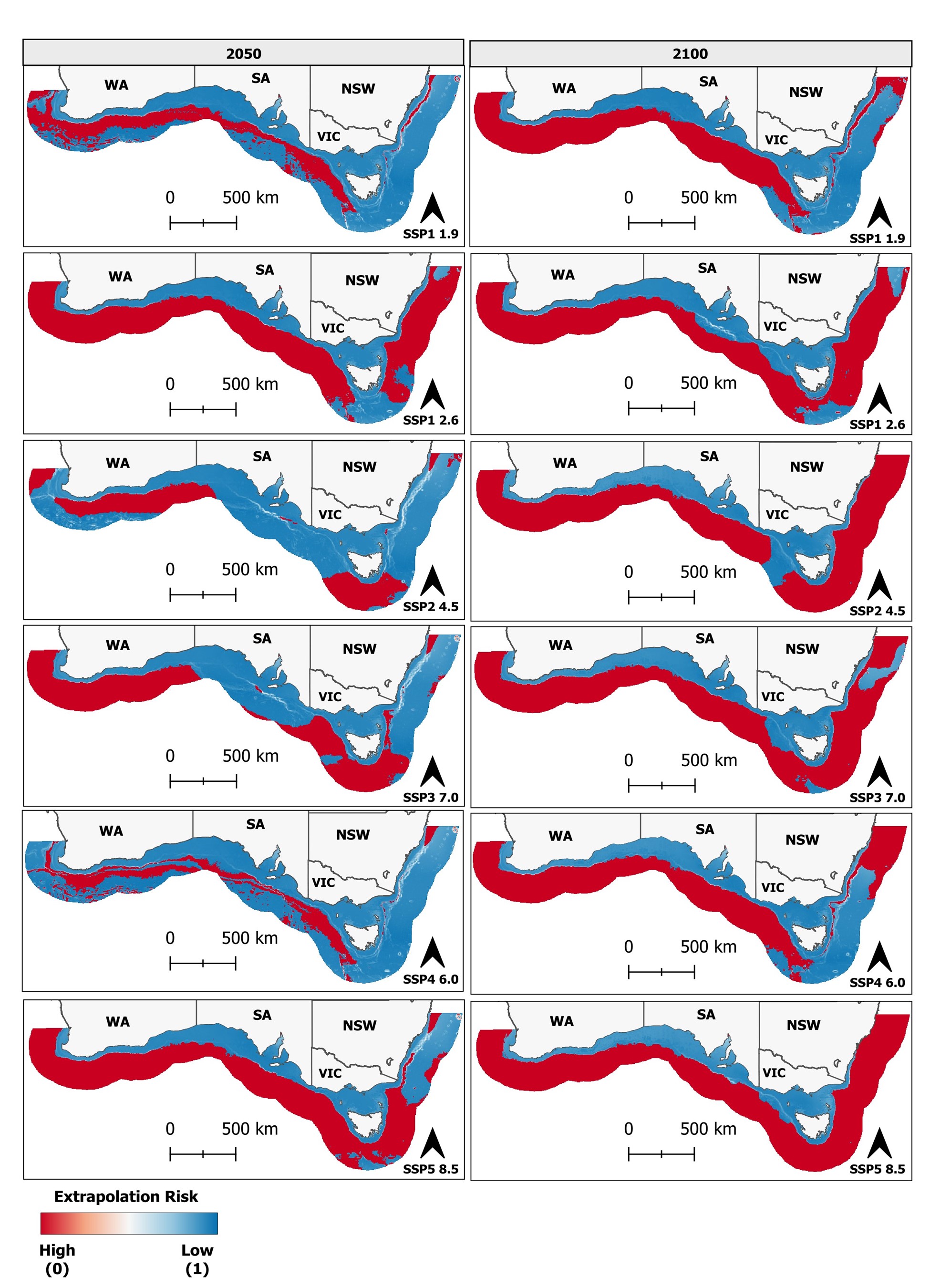

Supplement: Supplemental Information 2 — Red areas (value of zero) highlight areas of strict extrapolation; all other areas indicate the similarity between future conditions for 2050 (left panel) and 2100 (right panel) under SSP1-1.9, SSP1-2.6, SSP2-4.5, SSP3-7.0, SSP4-6.0 and SSP5-8.5 for the whitefin swellshark (Cephaloscyllium albipinnum). [file peerj-13-18787-s002.jpg]

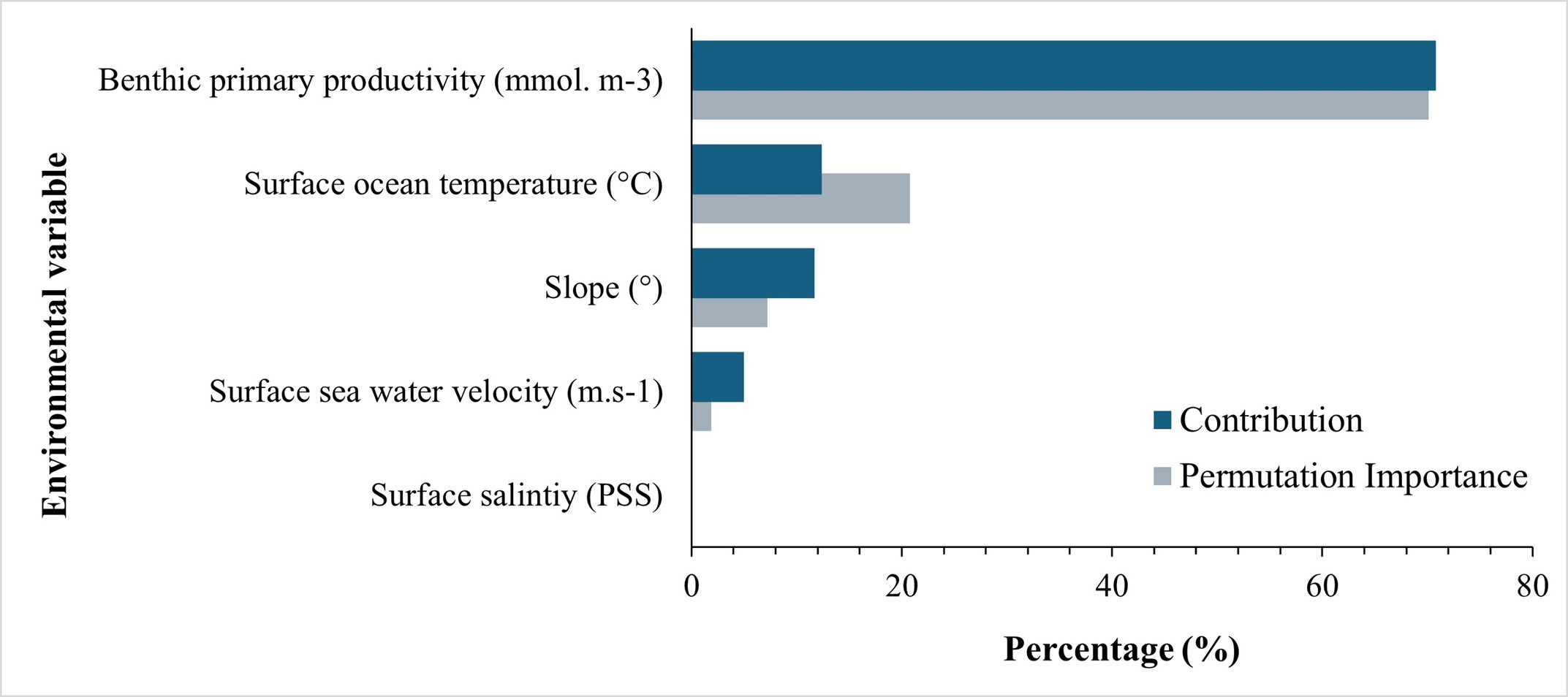

Supplement: Supplemental Information 3 — Values shown are averages over ten replicate runs. [file peerj-13-18787-s003.jpg]

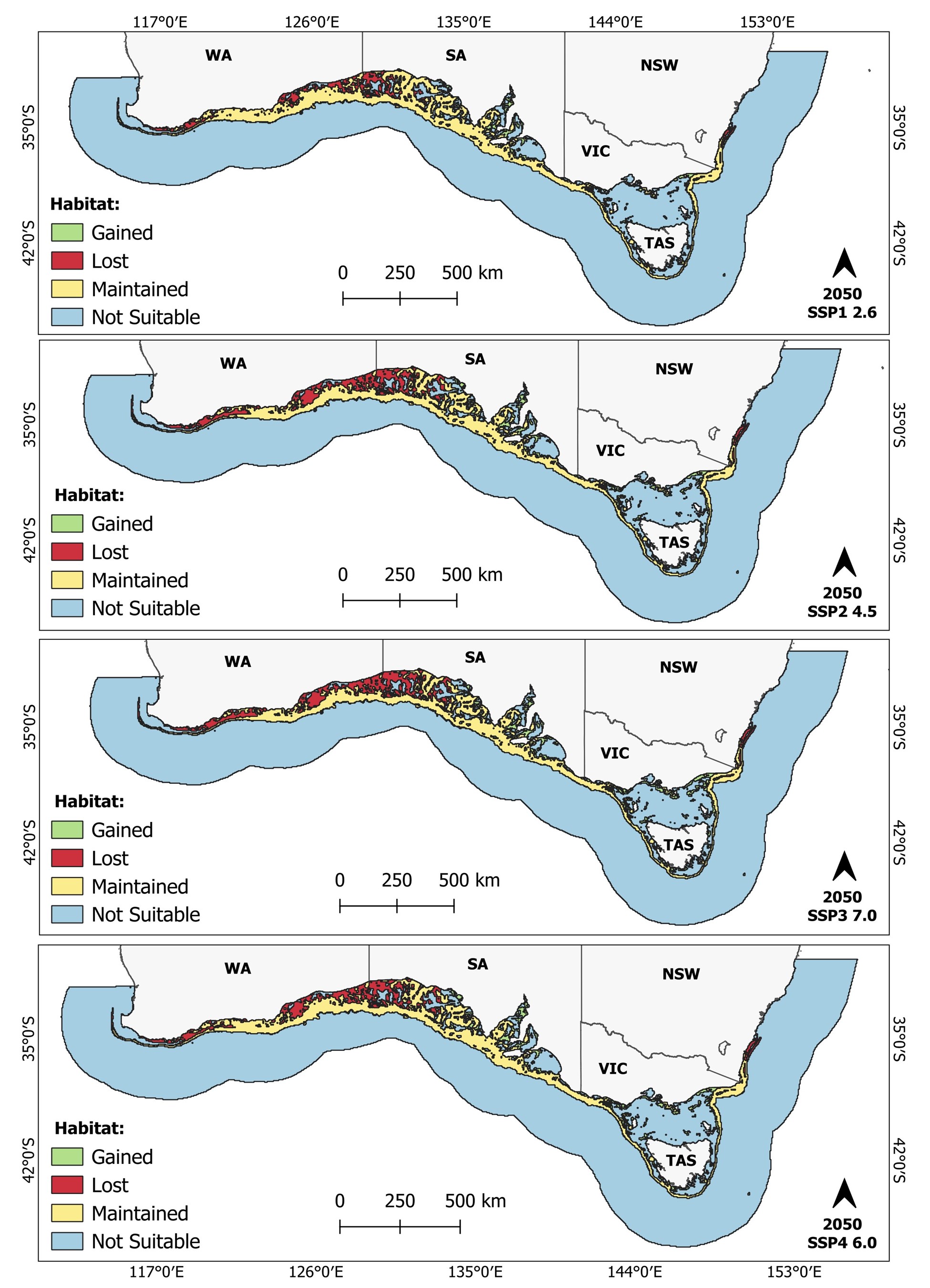

Supplement: Supplemental Information 4 — under scenarios SSP1-2.6 (top panel), SSP2-4.5 (second panel), SSP3-7.0 (third panel) and SSP4-6.0 (bottom panel) by the middle of the century (2040-2050). Unsuitable areas for C. albipinnum are shown in blue. Binary threshold = 0.3576. [file peerj-13-18787-s004.jpg]

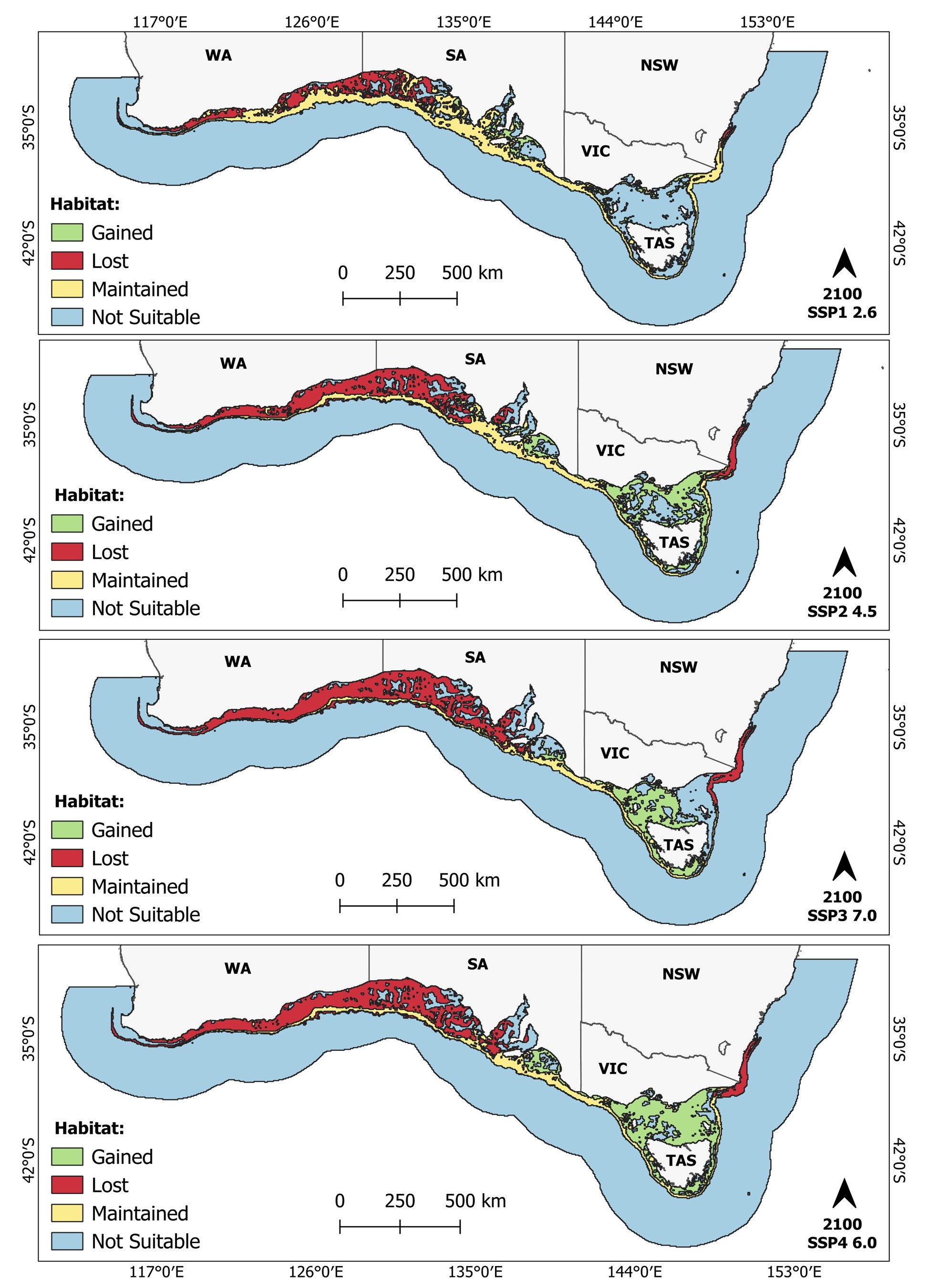

Supplement: Supplemental Information 5 — Suitable habitat changes under scenarios SSP1-2.6 (top panel), SSP2-4.5 (second panel), SSP3-7.0 (third panel) and SSP4-6.0 (bottom panel) by the end of the century (2090-2100). Unsuitable areas for C. albipinnum are shown in blue. Binary threshold = 0.3576. [file peerj-13-18787-s005.jpg]

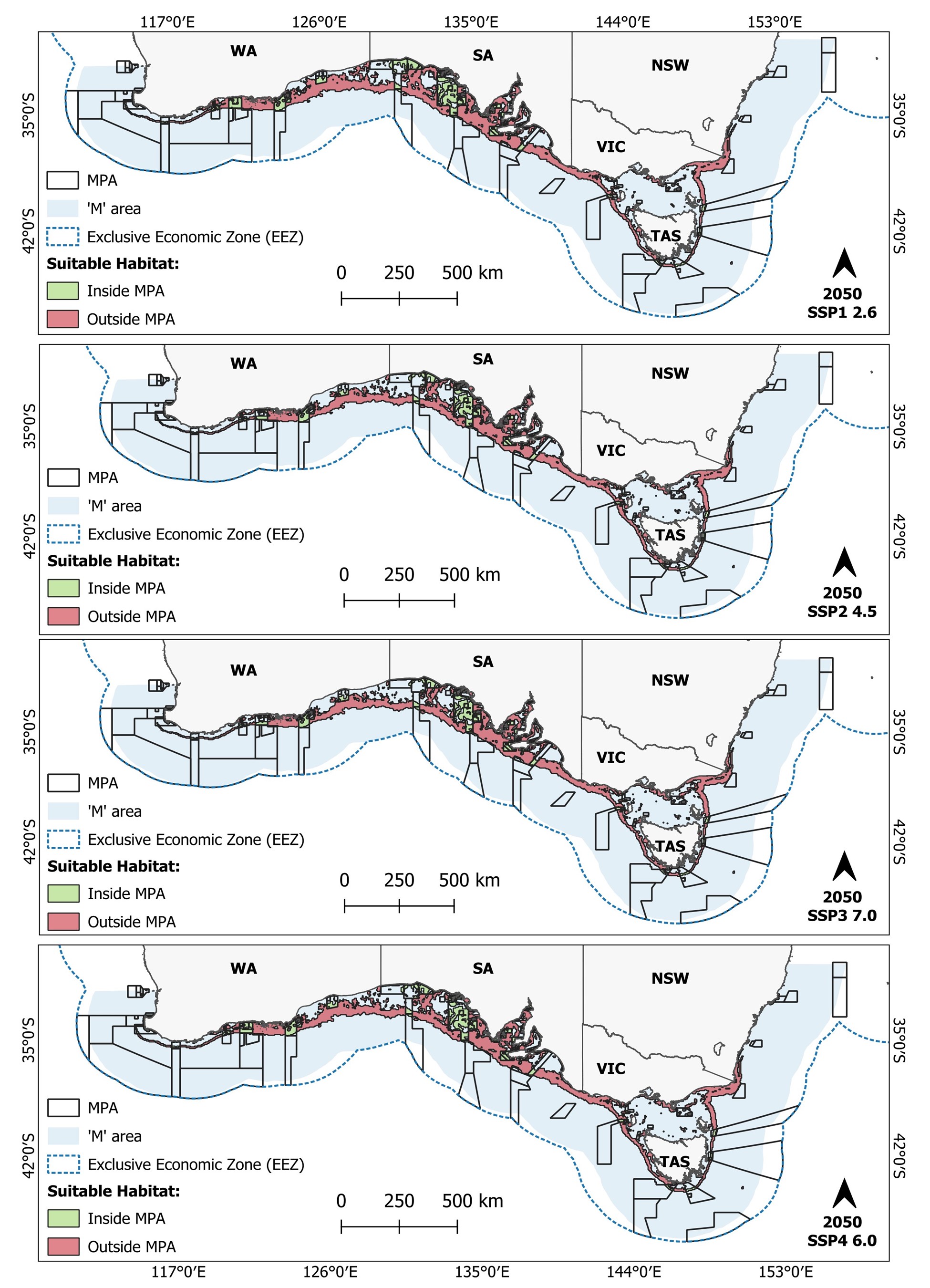

Supplement: Supplemental Information 6 — For scenarios SSP1-2.6 (top panel), SSP2-4.5 (second panel), SSP3-7.0 (third panel) and SSP4-6.0 (bottom panel) by the middle of the century (2040–2050). Binary threshold = 0.3576. [file peerj-13-18787-s006.jpg]

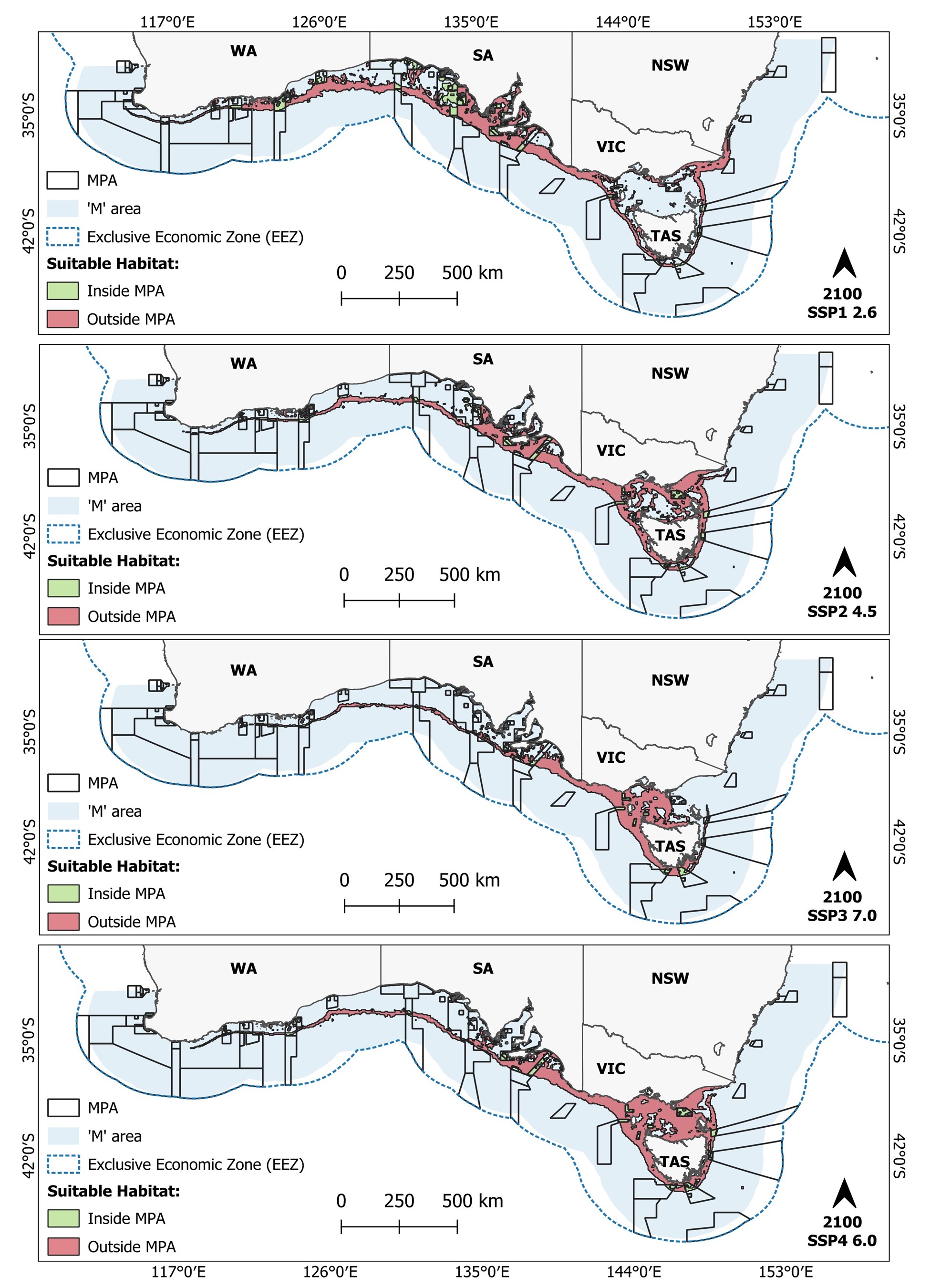

Supplement: Supplemental Information 7 — for scenarios SSP1-2.6 (top panel), SSP2-4.5 (second panel), SSP3-7.0 (third panel) and SSP4-6.0 (bottom panel) by the end of the century (2090–2100). Binary threshold = 0.3576. [file peerj-13-18787-s007.jpg]
