# Supplementary material for: Future climate-driven habitat loss and range shift of the Critically Endangered whitefin swellshark (Cephaloscyllium albipinnum)
Source: PeerJ. 2025 Feb 20;13:e18787. doi: 10.7717/peerj.18787 (PMC11847489; doi:10.7717/peerj.18787)
Supplement: Supplemental Information 8 [file peerj-13-18787-s008.pdf]

Variable	Unit	Source	Used in final model
Surface ocean temperature	°C	Bio-ORACLE (v3.0; Assis *et al.*, 2024)	Y
Benthic ocean temperature	°C	Bio-ORACLE (v3.0; Assis *et al.*, 2024)	N
Surface salinity	PSS	Bio-ORACLE (v3.0; Assis *et al.*, 2024)	Y
Benthic salinity	PSS	Bio-ORACLE (v3.0; Assis *et al.*, 2024)	N
Surface sea water velocity	$m\ s^{-1}$	Bio-ORACLE (v3.0; Assis *et al.*, 2024)	Y
Benthic sea water velocity	$m\ s^{-1}$	Bio-ORACLE (v3.0; Assis *et al.*, 2024)	N
Surface dissolved molecular oxygen	$nmol\ m^{-3}$	Bio-ORACLE (v3.0; Assis *et al.*, 2024)	N
Benthic dissolved molecular oxygen	$nmol\ m^{-3}$	Bio-ORACLE (v3.0; Assis *et al.*, 2024)	N
Surface primary productivity	$nmol\ m^{-3}$	Bio-ORACLE (v3.0; Assis *et al.*, 2024)	N
Benthic primary productivity	$nmol\ m^{-3}$	Bio-ORACLE (v3.0; Assis *et al.*, 2024)	Y
Surface chlorophyll	$nmol\ m^{-3}$	Bio-ORACLE (v3.0; Assis *et al.*, 2024)	N
Bathymetry	m	Bio-ORACLE (v3.0; Assis *et al.*, 2024)	N
Topographic slope	°	Bio-ORACLE (v3.0; Assis *et al.*, 2024)	Y
Substrate*	-	Commonwealth Scientific and Industrial Research Organisation (CSIRO) marine benthic substrate database (CSIRO, 2015)	N

* Substrate types: (1) biosiliceous marl and calcareous clay, (2) calcareous gravel, sand and silt, (3) calcareous ooze, (4) mud and calcareous clay, (5) mud and sand, (6) pelagic clay, (7) sand, silt and gravel with less than 50% mud, and (8) volcanic sand and grit.
