# Supplementary material for: Future climate-driven habitat loss and range shift of the Critically Endangered whitefin swellshark (Cephaloscyllium albipinnum)
Source: PeerJ. 2025 Feb 20;13:e18787. doi: 10.7717/peerj.18787 (PMC11847489; doi:10.7717/peerj.18787)
Supplement: Supplemental Information 9 [file peerj-13-18787-s009.pdf]

RM	FC	Mean AUC ratio	partial ROC	Omission rate 5%	AICc	ΔAICc	Weight AICc	N
4	h	1.717	0	0.047	2811.593	0	0.859	26

RM is the regularisation multiplier, FC is the feature class, N is the number of parameters
