# Supplementary material for: Future climate-driven habitat loss and range shift of the Critically Endangered whitefin swellshark (Cephaloscyllium albipinnum)
Source: PeerJ. 2025 Feb 20;13:e18787. doi: 10.7717/peerj.18787 (PMC11847489; doi:10.7717/peerj.18787)
Supplement: Supplemental Information 10 — By IUCN category, under SSP1-1.9, SSP1-2.6, SSP2-4.5, SSP3-7.0, SSP4-6.0 and SSP5-8.5 by the middle (2040–2050) and the end of the century (2090–2100) relative to the current (2010–2020) distribution. [file peerj-13-18787-s010.pdf]

Scenario	Suitable Habitat km^2					
	IUCN Ia	IUCN II	IUCN III	IUCN IV	IUCN V	IUCN VI
Current	2, 360	17, 853	3	6, 990	0	52, 178
**2050**						
SSP1 1.9	2, 180	16, 432	3	8, 594	0	51, 482
Change	-180	-1, 421	-	1, 604	-	-696
SSP1 2.6	1, 906	13, 694	3	9, 546	0	46, 724
Change	-454	-4, 159	-	2, 556	-	-5, 454
SSP2 4.5	1, 555	9, 498	3	9, 359	0	42, 164
Change	-805	-8, 355	-	2, 369	-	-10, 014
SSP3 7.0	1, 526	9, 114	3	9, 468	0	41, 180
Change	-834	-8, 739	-	2, 478	-	-10, 998
SSP4 6.0	1, 557	10, 879	3	9, 402	0	43, 485
Change	-1, 203	-6, 974	-	2, 412	-	-8, 693
SSP5 8.5	845	6, 082	3	9, 320	0	34, 640
Change	-1, 515	-11, 771	-	2, 330	-	-17, 538
**2100**						
SSP1 1.9	2, 324	17, 024	3	9, 107	0	54, 481
Change	-36	-829	-	2, 117	-	2, 303
SSP1 2.6	1, 341	7, 907	3	10, 565	0	38, 753
Change	-1, 019	-9, 946	-	3, 575	-	-13, 425
SSP2 4.5	229	2, 545	6	6, 206	4	25, 167
Change	-2, 131	-15, 308	3	-784	4	-27, 011
SSP3 7.0	0	1, 135	0	848	15	12, 378
Change	-2, 360	-16, 718	-3	-6, 142	15	-39, 800
SSP4 6.0	71	1, 793	0	3, 486	6	21, 198
Change	-2, 289	-16, 060	-3	-3, 504	6	-30, 980
SSP5 8.5	0	894	0	377	15	6, 618
Change	-2, 360	-16, 959	-3	-6, 613	15	-45, 560

Note: SSP = Shared Socioeconomic Pathway. IUCN Ia (strict nature reserve), II (national park), III (natural monument or feature), IV (habitat or species management area), V (protected seascape) and VI (protected area with sustainable use of natural resources).
